# Quantile regression analysis of modifiable and non-modifiable drivers’ of blood pressure among urban and rural women in Ghana

**DOI:** 10.1038/s41598-018-26991-4

**Published:** 2018-06-04

**Authors:** Dickson A. Amugsi, Zacharie T. Dimbuene, Gershim Asiki, Catherine Kyobutungi

**Affiliations:** 1 African Population and Health Research Center (APHRC), Nairobi, Kenya; 2 Department of Population Sciences and Development, University of Kinshasa, Kinshasa, Democratic Republic of the Congo; 3 Statistics Canada, Social Analysis and Modeling Division, Ottawa, K1A 0T6 Canada

## Abstract

High blood pressure is an increasingly problematic public health concern in many developing countries due to the associated cardiovascular and renal complications. This study set out to investigate the drivers of blood pressure among urban and rural women using the 2014 Ghana Demographic and Health Survey data. Diastolic blood pressure (DBP) and systolic blood pressure (SBP) were the outcomes of interest. Our findings showed that body mass index (BMI) had a significant positive effect on DBP and SBP in both urban and rural settings, with the largest effect occurring among women in the 75^th^ quantile. Arm circumference also had a positive effect on DBP and SBP across all quantiles in both settings. Age had an increasing positive effect along the entire conditional DBP and SBP distribution in both settings. Women who were pregnant had lower DBP and SBP relative to those who were not pregnant in both settings. These results highlight the important drivers of DBP and SBP, and the differential effects of these drivers on blood pressure (BP) among women in urban and rural settings. To increase their effectiveness, interventions to address high BP should take into account these differential effects.

## Introduction

High systolic and diastolic blood pressure are a global public health concern^1–3^. This is particularly the case owing to their contribution to the incidence of cardiovascular disease (CVD). A number of studies have found significant and linear relationships between SBP and DBP levels, and CVD morbidity and mortality^4,5^. Analysis of longitudinal data spanning 20 years confirms that SBP and DBP have continuous, graded, strong, independent, etiologically significant relationships to blood pressure-related risks, primarily incidence and mortality from coronary heart disease, stroke, and all CVDs^6^. These relationships were documented for young, middle-aged, and older men and women of varying socioeconomic backgrounds and ethnicities^6^, suggesting that the consequences of high DBP and SBP cut across all ages. Analysis of data from several national and regional surveys shows that diastolic and systolic hypertension is becoming more common in developing countries compared to developed countries^7,8^. It is estimated that by 2025, almost three-quarters of people with hypertension will be living in developing countries^8^. This high prevalence, coupled with poor hypertension management are important factors in the rising epidemic of CVDs in developing countries^7^.

Ghana, the focus of this study, has recently been classified among countries in the developing world with a high prevalence of diastolic and systolic hypertension^7^. Both urban and rural populations bear the brunt of a surge in hypertension, though the problem tends to be more serious among urban dwellers compared to their rural counterparts^9,10^. It is therefore imperative that we understand the drivers, both modifiable and non-modifiable, that contribute to increased diastolic and systolic hypertension in Ghana.

Several studies have observed that BMI is consistently and independently associated with DBP and SBP in women^9,11–16^. Kristjansson and colleagues^15^ observed that increases in BMI were associated with increases in DBP and SBP among urban women. A longitudinal analysis of the incidence of hypertension, revealed that a positive change in BMI was a significant predictor of hypertension, independent of other factors^15^. Further, overweight or obese individuals had higher odds of hypertension than those with normal BMI^17^. Consequently, most obese or overweight adults are hypertensive^16^. Evidence further reveals a strong relationship between arm circumference (AC) and BP (both DBP and SBP) in women, after accounting for important covariates such as age and gender^18^. Similarly, urban women with an AC > 33 cm had higher prevalence of high blood pressure than those with AC <33 cm^19^. In a another study, AC was found to be a strong independent predictor of SBP over time, but not DBP^20^.

Age is another driver of DBP and SBP. There is evidence that DBP and SBP increased with age^15^, with the rise in SBP tending to be greater than DBP^16^. A significant and positive correlation between age, and DBP and SBP was also observed in a related study in Northeast India^14^. A study in Ghana showed that, age was independently associated with SBP and DBP among rural women^11^. The rise in blood pressure with age has been attributed to multiple factors, including increased social stress, hardening and shrinking of the arteries, obesity and unhealthy lifestyle practices^21^. Besides the correlates discussed above, other factors such as education, employment, pregnancy, breastfeeding, salt intake, and socioeconomic status have been found to have varying effects on DBP and SBP among women^7,22–26^. Granted the variety and high number of drivers of DBP and SBP, investigation of these associations in developing countries, in the context of increasing incidence of CVD, is warranted.

The studies reviewed above either focus on urban areas, rural areas, or both. This may obscure commonalities and differences between urban and rural settings concerning the effects of the drivers of DBP and SBP. Understanding rural–urban differences might provide clues as to the most appropriate interventions to address the incidence of high BP in the two settings. Additionally, little is known about the differential effects of these drivers on DBP and SBP. This is partly due to methodological limitations. For example, studies mostly used either ordinary least squares (OLS) or logistic regression. These methods are popular in epidemiological studies but can only detect the average effect of the predictors on the outcomes; thereby, masking the conditional variations in the effects along the entire distribution of the outcome variable. The present study contributes to the existing literature by using quantile regression (QR) to examine the effects of the drivers along the entire distribution of the DBP and SBP in urban and rural settings. Findings from QR analysis provide a promising avenue to identify more vulnerable groups and devise more effective interventions for those at highest risk in urban and rural settings.

## Methodology

### Data sources and participants

The study used data from 2014 Ghana Demographic and Health Survey (GDHS)^27^. The survey was implemented by Ghana Statistical Service (GSS) and Ghana Health Service (GHS), with technical support from ICF International, USA. GDHS employs a two-stage probabilistic sampling design to select clusters, aimed to allow estimates of key indicators at national level, as well as for urban and rural areas in each of the 10 administrative regions in Ghana. The first stage involves selection of sample points (clusters) consisting of enumeration areas (EAs) from a master sampling frame constructed from the 2010 national population and housing census. A total of 427 clusters were selected, 216 in urban areas and 211 in rural areas^28^. The sampling frame excludes nomadic and institutional populations such as persons in hotels, barracks, and prisons^28^. The second stage involves a systematic selection of households within selected clusters. A household listing operation is then undertaken in all the selected EAs, and households to be included in the survey are randomly selected from the list. All women aged 15–49 in the selected households were eligible to be interviewed and have their blood pressure measured.

The household response rate was 99%, 98% for urban and 99% for rural. The response rate among eligible women was 97% and 98% in urban and rural settings respectively. The 2014 GDHS incorporated several biomarkers: blood pressure measurement, anthropometry, anaemia testing, and HIV testing. The present study focuses on the blood pressure (BP) measurements. The total eligible participants were 9,396; those with complete systolic and diastolic blood pressure data and used in the analysis was 9,352 (99.5%): 4,776 rural and 4,576 urban women respectively.

### Ethical considerations

The GDHS protocol, including biomarker collection, was reviewed and approved by the Ghana Health Service Ethical Review Committee and the Institutional Review Board of ICF International, USA. Written informed consent was obtained from participants before the survey. The GDHS team made sure that the biomarker results were made available to study participants. For example, the team made available results of BP measurements as well as information about the symptoms of high BP and ways in which it can be prevented through a BP reporting form. The DHS Program, USA, granted the authors permission to use the data. The data are completely anonymous; therefore the authors did not seek further ethical clearance.

## Measurements

### Outcomes

During the DHS individual interviews, three blood pressure measurements were taken from consenting women aged 15–49 in the selected households. Blood pressure was measured using the LIFE SOURCE® UA-767 Plus blood pressure monitor, a digital oscillometric blood pressure measuring device with automatic upper-arm inflation and automatic pressure release. Measurements were taken at intervals of 10 minutes or more^28^. For this analysis, the average of all the three measurements were used to create the systolic blood pressure (SBP) and diastolic blood pressure (DBP) variables^29,30^. Because QR was chosen as the analytical strategy, the two response variables were used in the analysis as continuous variables.

### Explanatory variables

The explanatory variables were classified into modifiable and non-modifiable factors. The modifiable factors included body mass index (BMI), number of years of education, salt intake (ate salted dried fish/koobi/kako added to food in the last 24 hours-yes/no), employment status (currently working-yes/no), fruits and vegetables consumption, arm circumference, exercise (exercised in the past 10 minutes-yes/no), pregnancy status (currently pregnant-yes/no or not sure), parity, breastfeeding (currently breastfeeding-yes/no), household wealth and household size. The wealth index in the DHS data set was created based on assets ownership and housing characteristics of each household: type of roofing, and flooring material, drinking water, sanitation facilities, ownership of television, bicycle and motorcycle, among others. Principal component analysis was employed to assign weights to each asset in each household. The asset scores were then summed up and individuals ranked according to the household score. The wealth index was then divided into quintiles: poorest, poorer, middle, richer and richest. The BMI was derived by dividing weight in kilograms by the square of height in meters. The weight measurements were undertaken using electronic Seca scales with a digital screen, which was designed and produced under the guidance of UNICEF. Height measurements were obtained using stadiometer. The AC measurement was taken from the mid-upper arm of the study participants using a graduated measuring tape. Age was the only non-modifiable factor included in the analysis. The potential drivers of women BP as outlined above were identified in the literature and subjected to bivariate analysis to establish which variables were significantly related to the BP measures. All significant variables in bivariate analyses were included in the multivariate analysis. Additionally, variables that were not significant but were considered critical by the researchers were included in the analysis. Such variables included, education, salt intake, and fruits and vegetables consumption.

## Analytical approach

We used quantile regression (QR)^31^ to estimate the effects of putative factors on systolic and diastolic blood pressure. The QR was introduced by Koenker and Bassett^31^ as a location model to extend Ordinary Least Squares (OLS), which summarizes the distribution at its grand mean to a more general class of linear models. In this regard, the conditional quantiles have linear form to fully account for the overall distribution of the response variable. Statistical analyses included descriptive and multivariate QR techniques using STATA version 14. The descriptive analyses explored the characteristics of the sample. The multivariate analysis explored the relationship between the predictor variables and the outcome variable. Employing QR to conduct the multivariate analysis made it possible to capture the full distribution of the outcome variable. We estimated ordinary least square (OLS) regressions and quantile regressions (QR) at the 5^th^, 10^th^, 25^th^, 50^th^, 75^th^ and 90^th^ quantiles^32,33^. We chose QR over OLS regression to examine the effects of the predictor variables at different points of conditional distribution of the outcome variables (SBP and DBP). This cannot be done with OLS, because standard OLS regression techniques summarize the average relationship between a set of regressors and the outcome variable based on the conditional mean function E (y|x). This provides only a partial view of the relationship, as we might be interested in describing the relationship at different points in the conditional distribution of y. Thus, the QR unlike OLS provides a more complete view of the effect of the explanatory variables on the outcome. Therefore it is possible to identify which groups are more vulnerable and need intervening.

The QR analysies were stratified by place of residence (urban-rural) to aid in understanding the drivers in the two settings. For QR estimates, since the study was interested in all potential drivers of BP, independent variables, including (BMI), number of years of education, age in years, salted dried fish and fruits consumption, employment status, arm circumference, exercise status, pregnancy status, parity, breastfeeding status, household wealth and household size were included in the models at the same time. Since we were also interested in the differences between OLS and QR estimates, the OLS results were generated.

## Results

### Descriptive analysis of the characteristics of the sample

Table 1 presents the characteristics of the urban and rural samples used in the analysis. The results showed that the mean DBP and SBP were higher among women in the urban compared to women in rural settings. The BMI of urban women was also higher than the BMI of rural women (26.03 ± 5.79 *vs*. 23.58 ± 4.34). A similar trend was observed with AC. Parity was higher among rural women relative to urban women (2.84 ± 2.59 *vs*.1.94 ± 2.10), and consequently, household sizes were bigger in the rural settings. Urban women had more years of education (8.37 ± 4.17 *vs*.5.49 ± 4.28). Mean ages for women in the rural and urban settings were fairly similar (30.02 ± 9.37 vs 29 ± 9.85).

**Table 1:** Characteristics of the Sample.

Variable	Urban		Rural	
	Mean	SD	Mean	SD
Systolic blood pressure	113.39	17.10	110.22	15.38
Diastolic blood pressure	75.97	11.84	72.89	10.86
BMI (Kg/m^2^)	26.03	5.79	23.58	4.34
Age (in years)	30.02	9.37	29.68	9.85
Education (in years)	8.37	4.17	5.49	4.28
Children ever born (parity)	1.94	2.10	2.84	2.59
Household size	4.41	2.32	5.57	3.10
Arm circumference (cm)	30.35	4.95	28.64	4.18

SD = Standard deviation.

### Multivariate analysis of the rural sample

Tables 2 and 3 present the results of modifiable and non-modifiable drivers of DBP and SBP in rural settings respectively. OLS estimates indicated a strong positive effect of BMI, age, and AC on DBP and SBP. However, there was an inverse relationship between DBP and SBP and parity, pregnancy, and breastfeeding status.

**Table 2:** Multivariate quantile regression analysis of drivers of Diastolic blood pressure among Rural women.

	OLS	Q 5	Q 10	Q 25	Q 50	Q 75	Q 90
**Variable**							
BMI (Kg/m^2^)	0.192***	0.085	0.206*	0.198**	0.218***	0.230**	0.106
	(0.054)	(0.087)	(0.093)	(0.072)	(0.064)	(0.078)	(0.135)
Age (in years)	0.300***	0.155***	0.114**	0.168***	0.270***	0.360***	0.519***
	(0.027)	(0.040)	(0.042)	(0.034)	(0.038)	(0.043)	(0.055)
Is Working (yes)	−0.353	−0.129	−0.110	−0.382	−0.515	−0.244	−1.171
	(0.359)	(0.595)	(0.543)	(0.419)	(0.386)	(0.487)	(0.620)
Ate Salted Fish (yes)	0.547	−0.626	0.179	0.299	0.442	0.550	1.120*
	(0.299)	(0.507)	(0.446)	(0.359)	(0.346)	(0.401)	(0.549)
Ate Fruits in last 7 days (yes)	−4.307	−5.929	−3.605	−3.768	−7.468	−6.722	−3.537
	(3.952)	(4.298)	(4.153)	(4.581)	(4.757)	(4.850)	(3.833)
Ate Vegetables in last 7 days (yes)	0.017	0.195*	0.163*	0.133*	−0.041	−0.022	−0.034
	(0.055)	(0.098)	(0.074)	(0.064)	(0.062)	(0.075)	(0.112)
Education (in years)	0.083*	−0.065	−0.006	0.010	0.119*	0.137*	0.126
	(0.042)	(0.068)	(0.058)	(0.054)	(0.055)	(0.059)	(0.090)
**Wealth index (poorest ref)**							
Wealth index (poor)	0.508	0.626	0.132	0.371	0.670	0.348	0.930
	(0.358)	(0.608)	(0.504)	(0.432)	(0.406)	(0.532)	(0.707)
Wealth index (middle)	0.422	0.400	−0.240	0.304	0.331	0.221	1.197
	(0.438)	(0.839)	(0.538)	(0.514)	(0.605)	(0.599)	(0.878)
Wealth index (rich)	0.566	0.647	1.286	0.937	1.194	−0.008	1.393
	(0.644)	(1.312)	(1.139)	(0.806)	(0.739)	(0.999)	(1.285)
Wealth index (richest)	0.830	3.421*	1.341	0.267	3.448*	−2.012	−2.256
	(1.793)	(1.508)	(1.921)	(3.453)	(1.544)	(1.719)	(2.405)
Children ever born (parity)	−0.297**	−0.132	−0.080	−0.138	−0.285*	−0.429**	−0.394
	(0.100)	(0.154)	(0.148)	(0.125)	(0.143)	(0.157)	(0.210)
Is Pregnant (yes)	−6.290***	−5.286***	−5.308***	−5.326***	−5.980***	−7.741***	−8.190***
	(0.533)	(0.741)	(0.783)	(0.613)	(0.604)	(0.639)	(1.146)
Is Breastfeeding (yes)	−0.744*	−1.590**	−1.231*	−0.799	−0.600	−1.123*	−1.009
	(0.339)	(0.614)	(0.497)	(0.465)	(0.395)	(0.459)	(0.645)
Household size	−0.033	0.056	0.024	−0.031	0.050	−0.041	−0.102
	(0.050)	(0.065)	(0.070)	(0.061)	(0.063)	(0.065)	(0.082)
Arm circumference	0.437***	0.432***	0.432***	0.435***	0.357***	0.414***	0.458***
	(0.045)	(0.090)	(0.062)	(0.069)	(0.068)	(0.077)	(0.076)
Exercise in last 10 mins (yes)	0.319	0.389	0.261	−0.022	−0.325	0.384	1.183
	(0.422)	(0.738)	(0.536)	(0.479)	(0.465)	(0.607)	(1.113)
Observations	4776	4776	4776	4776	4776	4776	4776

Standard errors in parentheses; Q = Quantile; OLS = ordinary least square.

*p < 0.05 ** p < 0.01 ***p < 0.001.

**Table 3:** Multivariate quantile regression analysis of drivers of systolic blood pressure among rural women.

	OLS	Q 5	Q 10	Q 25	Q 50	Q 75	Q 90
**Variable**							
BMI (Kg/m^2^)	0.174*	0.132	0.262**	0.234**	0.215*	0.080	0.131
	(0.079)	(0.120)	(0.082)	(0.072)	(0.094)	(0.112)	(0.195)
Age (in years)	0.440***	0.067	0.062	0.108**	0.284***	0.490***	0.901***
	(0.039)	(0.054)	(0.046)	(0.041)	(0.052)	(0.064)	(0.096)
Is Working (yes)	−1.400**	0.117	−0.273	−0.122	−1.097*	−1.720**	−2.981**
	(0.519)	(0.762)	(0.566)	(0.488)	(0.517)	(0.628)	(0.967)
Ate Salted Fish (yes)	0.594	−0.163	0.278	0.619	0.750	0.648	0.114
	(0.432)	(0.630)	(0.490)	(0.433)	(0.445)	(0.599)	(0.879)
Ate Fruits in last 7 days (yes)	−2.438*	−10.305***	−7.707**	−5.555	−3.301	2.341	−2.124
	(5.710)	(2.677)	(2.504)	(2.958)	(3.367)	(5.599)	(7.154)
Ate Vegetables in last 7 days (yes)	−0.019	−0.003	−0.018	0.089	0.007	−0.014	0.029
	(0.080)	(0.108)	(0.086)	(0.075)	(0.081)	(0.126)	(0.165)
Education (in years)	0.088	0.043	0.030	0.085	0.120	0.141	0.004
	(0.060)	(0.080)	(0.075)	(0.059)	(0.062)	(0.083)	(0.134)
**Wealth index (poorest ref)**							
Wealth index (poor)	1.174*	0.161	0.183	0.474	1.041*	1.139	2.287
	(0.517)	(0.694)	(0.602)	(0.474)	(0.490)	(0.683)	(1.197)
Wealth index (middle)	1.234	−0.409	−0.151	0.043	0.669	1.433	2.741
	(0.633)	(1.033)	(0.774)	(0.637)	(0.664)	(1.019)	(1.405)
Wealth index (rich)	−0.096	0.134	−0.429	−0.569	0.170	−0.766	5.856*
	(0.930)	(1.349)	(0.971)	(0.980)	(1.060)	(1.290)	(2.488)
Wealth index (richest)	−0.630	−4.278	0.587	0.433	−0.911	−1.544	−7.450
	(2.592)	(4.033)	(4.437)	(2.726)	(3.232)	(2.692)	(4.689)
Children ever born (parity)	−0.573***	−0.403*	−0.388**	−0.294	−0.506**	−0.572*	−1.113**
	(0.145)	(0.200)	(0.136)	(0.167)	(0.173)	(0.234)	(0.341)
Is Pregnant (yes)	−6.259***	−3.348***	−4.415***	−5.077***	−5.656***	−7.184***	−9.444***
	(0.771)	(0.960)	(0.614)	(0.782)	(0.711)	(0.772)	(1.275)
Is Breastfeeding (yes)	−1.573**	−0.644	−0.344	−0.861	−1.523**	−2.164**	−3.342***
	(0.490)	(0.726)	(0.542)	(0.483)	(0.502)	(0.670)	(0.998)
Household Size	−0.025	0.120	0.050	0.047	0.044	0.007	0.017
	(0.073)	(0.089)	(0.066)	(0.070)	(0.064)	(0.100)	(0.123)
Arm circumference	0.553***	0.572***	0.572***	0.534***	0.508***	0.558***	0.505***
	(0.064)	(0.090)	(0.084)	(0.074)	(0.083)	(0.103)	(0.134)
Exercise in last 10 mins (yes)	1.368*	0.419	1.154	0.268	0.532	1.896	2.781
	(0.609)	(1.085)	(0.827)	(0.577)	(0.676)	(1.053)	(1.780)
Observations	4776	4776	4776	4776	4776	4776	4776

Standard errors in parentheses; Q = Quantile; OLS = ordinary least square.

*p < 0.05 **p < 0.01 ***p < 0.001.

In multivariate analysis, the effect of BMI on DBP was observed at the 10^th^, 25^th^ 50^th^ and 75^th^ quantiles, with the largest effect occurring at the 75^th^ quantile. However, the effects of BMI on SBP were significant at 10^th^, 25^th^ and 50^th^ quantiles. There was an increasing effect of maternal age on DBP across all quantiles, with the least effect occurring at the 10^th^ quantile and the largest effect at the upper most quantile (90^th^). A year increase in age was associated with an increase of 0.114 and 0.519 in DBP at 10^th^ and 90^th^ quantiles respectively. Women’s age was associated with an increase in SBP at the 25^th^, 50^th^ 75^th^ and 90^th^ quantiles, with the largest effect occurring at the 90^th^ quantiles. Arm circumference had significant and positive effects on DBP and SBP across all quantiles, with the largest effect occurring at the upper most quantile (90^th^) in the case of DBP and the first two lower quantiles (5^th^ and 10^th^) in the case of SBP. Conversely, parity was negatively associated with DBP at 50^th^ and 75^th^ quantiles. An additional birth was associated with a 0.285 and 0.429 decrease in DBP at 50^th^ and 75^th^ quantiles respectively. On the other hand, the negative effect of parity on SBP was throughout the conditional SBP distribution (5^th^, 10^th^, 25^th^, 50^th^, 75^th^, and 90^th^ quantiles), with the largest decrease occurring at the 25^th^ quantile and least occurring at the 90^th^ quantile. The findings also indicated that being pregnant was negatively associated with DBP and SBP across all quantiles. An inverse relationship was observed between breastfeeding and DBP at the 5^th^, 10^th^ and 75^th^ quantiles, while the negative association occurred at the 50^th^, 75^th^ and 90^th^ quantiles in the case of SBP. Thus, women who were breastfeeding and were at these quantiles were likely to have lower SBP relative to those who were not breastfeeding.

### Multivariate analysis of the urban sample

Tables 4 and 5 present the results of the drivers of diastolic and systolic blood pressure in urban settings respectively. For comparison, OLS estimates are also reported. OLS estimates indicated a strong body mass index (BMI) effect on DBP and SBP, with both increasing with BMI. DBP and SBP increased with age, while both outcomes declined in women who were pregnant. Arm circumference (AC) was also positively associated with DBP and SBP. There was an inverse relationship between pregnancy and DPB and SBP, with pregnant women having lower blood pressure relative to those who were not pregnant.

**Table 4:** Multivariate quantile regression analysis of drivers of diastolic blood pressure among urban women.

	OLS	Q 5	Q 10	Q 25	Q 50	Q 75	Q 90
**Variable**							
BMI (Kg/m^2^)	0.261***	−0.025	0.065	0.254***	0.271***	0.361***	0.253
	(0.049)	(0.099)	(0.065)	(0.070)	(0.052)	(0.077)	(0.140)
Age (in years)	0.368***	0.256***	0.299***	0.282***	0.343***	0.377***	0.480***
	(0.028)	(0.044)	(0.041)	(0.035)	(0.033)	(0.043)	(0.067)
Is Working (yes)	−0.343	0.780	0.042	−0.423	−0.424	0.103	−0.378
	(0.373)	(0.651)	(0.508)	(0.367)	(0.358)	(0.488)	(0.686)
Ate Salted Fish (yes)	−0.236	0.272	−0.019	−0.349	−0.147	−0.416	−0.187
	(0.324)	(0.555)	(0.461)	(0.353)	(0.332)	(0.460)	(0.702)
Ate fruits in last 7 days (yes)	4.553	−5.057	−1.574	4.485	2.769	5.839	12.382***
	(4.590)	(3.306)	(3.447)	(4.642)	(5.375)	(4.633)	(3.364)
Ate vegetables in last 7 days (yes)	−0.009	−0.006	−0.077	0.022	−0.110	0.039	−0.043
	(0.059)	(0.098)	(0.086)	(0.062)	(0.061)	(0.077)	(0.127)
Education (in years)	0.039	0.116	0.057	0.018	0.039	−0.014	−0.000
	(0.042)	(0.076)	(0.060)	(0.049)	(0.041)	(0.058)	(0.095)
**Wealth index (poorest ref)**							
Wealth index (poor)	1.173	−0.803	−0.373	0.878	1.586	3.109*	3.571*
	(0.839)	(1.044)	(1.161)	(0.828)	(0.904)	(1.224)	(1.743)
Wealth index (middle)	2.034**	−0.843	0.508	1.537*	1.556	2.830**	3.454*
	(0.733)	(0.997)	(1.008)	(0.697)	(0.799)	(1.009)	(1.552)
Wealth index (rich)	1.815*	−0.198	0.583	1.565*	1.092	2.434*	2.503
	(0.726)	(0.941)	(0.953)	(0.669)	(0.770)	(0.959)	(1.529)
Wealth index (richest)	1.895*	−1.201	0.306	1.426*	1.295	2.877**	4.227**
	(0.751)	(1.051)	(0.991)	(0.712)	(0.806)	(1.045)	(1.607)
Children ever born (parity)	−0.038	−0.515*	−0.424*	−0.215	−0.249	0.099	0.455
	(0.124)	(0.209)	(0.180)	(0.170)	(0.137)	(0.203)	(0.274)
Is Pregnant (yes)	−6.424***	−4.807***	−5.477***	−5.474***	−6.633***	−6.130***	−5.932***
	(0.616)	(1.167)	(0.841)	(0.634)	(0.670)	(0.935)	(1.062)
Is Breastfeeding (yes)	−0.550*	−0.984	−0.869	−0.971*	−0.956*	−0.102	−0.365
	(0.423)	(0.686)	(0.578)	(0.443)	(0.455)	(0.627)	(0.931)
Household Size	−0.060	−0.020	−0.003	0.040	−0.038	−0.118	−0.163
	(0.070)	(0.126)	(0.098)	(0.074)	(0.064)	(0.094)	(0.129)
Arm circumference (cm)	0.317***	0.469***	0.382***	0.318***	0.331***	0.229***	0.357***
	(0.041)	(0.076)	(0.061)	(0.045)	(0.045)	(0.053)	(0.093)
Exercise in last 10 mins (yes)	−0.249	−1.372	−1.198	−0.113	−0.006	0.048	0.090
	(0.520)	(0.923)	(0.863)	(0.609)	(0.530)	(0.655)	(1.223)
Observations	4576	4576	4576	4576	4576	4576	4576

Standard errors in parentheses; Q = Quantile; OLS = ordinary least square.

*p < 0.05 **p < 0.01 ***p < 0.001.

**Table 5:** Multivariate quantile regression analysis of drivers of systolic blood pressure among urban women.

	OLS	Q 5	Q 10	Q 25	Q 50	Q 75	Q 90
**Variable**							
BMI (Kg/m^2^)	0.308***	0.150	0.232**	0.302***	0.361***	0.389***	0.267
	(0.073)	(0.102)	(0.090)	(0.074)	(0.086)	(0.105)	(0.194)
Age (in years)	0.565***	0.166**	0.179**	0.264***	0.392***	0.623***	1.029***
	(0.042)	(0.058)	(0.056)	(0.039)	(0.048)	(0.063)	(0.120)
Is Working (yes)	−1.155*	0.470	0.456	−0.646	−0.521	−1.610*	−2.970**
	(0.551)	(0.650)	(0.679)	(0.442)	(0.554)	(0.636)	(1.050)
Ate Salted Fish (yes)	−0.107	−0.687	0.101	−0.262	−0.260	−0.274	0.416
	(0.480)	(0.666)	(0.551)	(0.407)	(0.448)	(0.621)	(1.011)
Ate fruits in last 7 days (yes)	1.755	−3.753	−0.320	5.936	−0.713	2.697	3.693
	(6.786)	(6.182)	(6.494)	(8.439)	(10.334)	(10.210)	(8.248)
Ate vegetables in last 7 days (yes)	0.009	0.041	0.031	0.041	−0.042	−0.103	0.045
	(0.087)	(0.111)	(0.104)	(0.080)	(0.084)	(0.114)	(0.175)
Education (in years)	−0.057	0.006	−0.050	−0.054	−0.072	−0.191*	−0.157
	(0.062)	(0.080)	(0.065)	(0.059)	(0.057)	(0.089)	(0.137)
**Wealth index (poorest ref)**							
Wealth index (poor)	1.333	1.344	1.789	2.218	1.281	1.493	4.482*
	(1.240)	(1.541)	(1.133)	(1.178)	(1.413)	(1.648)	(1.846)
Wealth index (middle)	2.705*	1.349	2.043	3.161**	2.184	1.879	5.426**
	(1.083)	(1.327)	(1.067)	(1.116)	(1.292)	(1.257)	(1.698)
Wealth index (rich)	2.681*	1.051	1.858	2.771**	1.775	1.304	5.457***
	(1.074)	(1.232)	(1.078)	(1.041)	(1.388)	(1.265)	(1.622)
Wealth index (richest)	2.591*	0.639	1.353	2.667*	1.935	1.977	5.776***
	(1.110)	(1.307)	(1.115)	(1.064)	(1.447)	(1.300)	(1.695)
Children ever born (parity)	−0.191	−0.514*	−0.596**	−0.387	−0.446*	−0.213	0.326
	(0.184)	(0.241)	(0.224)	(0.209)	(0.203)	(0.340)	(0.513)
Is Pregnant (yes)	−7.210***	−2.937***	−4.147***	−5.531***	−5.652***	−7.918***	−10.159***
	(0.910)	(0.840)	(0.968)	(0.747)	(0.659)	(0.942)	(1.259)
Is Breastfeeding (yes)	−0.907	−0.082	−0.594	−1.472*	−0.610	0.151	−1.551
	(0.625)	(0.805)	(0.625)	(0.651)	(0.741)	(0.891)	(1.326)
Household Size	0.049	0.034	−0.154	−0.071	−0.007	0.103	0.275
	(0.103)	(0.122)	(0.135)	(0.081)	(0.104)	(0.120)	(0.226)
Arm circumference (cm)	0.313***	0.325***	0.349***	0.318***	0.287***	0.273***	0.318*
	(0.061)	(0.076)	(0.073)	(0.056)	(0.063)	(0.079)	(0.155)
Exercise in last 10 mins (yes)	0.902	1.021	0.637	−0.195	1.690*	0.760	3.057
	(0.768)	(0.954)	(0.791)	(0.616)	(0.846)	(0.924)	(2.044)
Observations	4577	4577	4577	4577	4577	4577	4577

Standard errors in parentheses; Q = Quantile; OLS = ordinary least square.

*p < 0.05 **p < 0.01 ***p < 0.001.

The quantile regression results show important differences in the conditional distributions of DBP and SBP. The effect of BMI on DBP was significant at the 25^th^, 50^th^ and 75^th^ quantiles. The largest effect occurred at the 75^th^ quantile. For SBP, significant effects were found at the 10^th^, 25^th^, 50^th^ and 75^th^ quantiles, with the largest effect at the 75^th^ quantile. A unit increase in BMI was associated with 0.232, 0.302, 0.361 and 0.389 increase in SBP at 10^th^, 25^th^, 50^th^ and 75^th^ quantiles respectively. As age increased, both DBP and SBP significantly increased across all quantiles, with the least effect at the lowest quantile (5^th^) and largest effect at the upper most quantile (90^th^) in both outcomes. A similar trend was observed for AC, where the effect was positive across all quantiles, with the largest effect at the 5^th^ quantile in the case of DBP and at the 10^th^ quantile in the case of SBP. Conversely, pregnancy was inversely related to both DBP and SBP and across all quantiles. There was also a negative association between breastfeeding and DBP at 25^th^ and 50^th^ quantiles.

## Discussion

This paper investigated modifiable and non-modifiable drivers of DBP and SBP among women in Ghana, using quantile regression to elucidate the differential effects of each putative driver on blood pressure. The results showed that BMI had a positive effect on DBP among urban women at three quantiles (25^th^, 50^th^ and 75^th^), and four quantiles (10^th^, 25^th^, 50^th^ and 75^th^) in the case of SBP. In rural settings, there was a positive effect of BMI on DBP at four quantiles, and on SBP at three quantiles (10^th^, 25^th^ and 50^th^). The largest effect occurred at the 75^th^ quantile of the conditional DBP and SBP distribution in urban settings and DBP in rural settings. These findings suggest that women at the 75^th^ quantile and who are likely to be at risk of high blood pressure may benefit more from the reduction of women BMI than those in the lower end of the distribution. Differences in the effects of BMI on women DBP and SBP are contrary to OLS results, which misleadingly indicated that BMI had significant positive effect on blood pressure of all women in urban and rural settings respectively. Arm circumference also had a strong positive effect on DBP and SBP across all quantiles in urban and rural settings. This suggests that anthropometry plays a critical role in driving women BP.

These findings are in line with previous research. A study conducted in Africa and Asia observed a positive association between BMI, and DBP and SBP, and the risk of hypertension was high among population groups with higher BMI^13^. Studies in Ghana also documented BMI as an independent predictor of SBP and SBP^9,11,34^. BMI was independently associated with BP in rural and urban settings in Ghana^34^. The limitation of the above studies is that they all investigated the average effect of BMI on women systolic and diastolic BP. It is therefore important to point out that the findings of these studies are consistent with OLS results obtained in the present study but only partly consistent with the QR results, as the QR results show differential effects of BMI along the conditional DBP and SBP distributions. Along with previous research, findings from the present study clearly indicate the important contribution of BMI to blood pressure among women. Interventions to address BMI might have a positive effect on blood pressure.

Age is another important driver of DBP and SBP among urban and rural women in Ghana. Our results show that age is significantly and positively associated with DBP and SBP in Ghana. The effect increases along the entire conditional distribution of the DBP and SBP in urban settings. This suggests that younger women (at lower tail of distribution) experienced the least effect, while older women (at upper tail of the distribution) experienced the largest effect. However, in rural settings, increasing effects of age along the entire distribution of women BP was limited to DBP, while age was associated with increase in SBP at 25^th^, 50^th^, 75^th^ and 90^th^ quantiles. Like urban settings, the largest effect of age on rural women’s DBP and SBP was at the upper tail (90^th^) of the conditional distribution of the two outcomes. This suggests that effects of age on DBP and SBP accrue disproportionately to women in the upper tail of the conditional DBP and SBP distributions, and who are at higher risks of hypertension. The analysis clearly show that as age increases, blood pressure also increases, irrespective of whether one lives in urban or rural settings. This is not unexpected since age is a non-modifiable factor and not affected by the environmental changes. However, the OLS results show that age had a strong effect, with DBP and SBP increasing equally with age for all women, which paints just a part of the picture and can be misleading. The findings of our studies are similar to findings of other studies in the literature. Several studies using either logistic regression or OLS have shown that age associates positively with DBP and SBP in women^2,3,9,11,15,35^. In Ghana, age was independently associated with DBP and SBP in women^9,11^. Another study revealed that BP increased significantly with increasing age in both rural and urban populations^3^. The literature together with the present study illuminated the importance of age as a driver of BP among women in urban and rural settings.

Unexpectedly, being pregnant was inversely associated with women systolic and diastolic BP in both urban and rural settings and across all quantiles. Women who were pregnant at the time of the survey tended to have lower DBP and SBP compared to those that were not. The largest effect in both outcomes is at the lowest quantile (5^th^). This is contrary to belief, supported by some studies, that pregnancy induces high BP^36,37^. For example, a recent study showed that mean DBP and SBP levels were consistently higher during the first pregnancy at comparable weeks of gestation, significantly so during the third trimester^36^. However, another study suggested that whether pregnancy will induce high BP or not depended to a large extend on maternal pre-gestation BMI: a positive association was reported between maternal pre-gestational BMI and mean systolic and diastolic blood pressure^38^. It is important to note that some other studies have observed an inverse relationship between pregnancy and BP at certain stages of the pregnancy. Iwasaki and colleagues^39^ observed that women with high initial blood pressure tended to exhibit a fall in blood pressure, whereas women with low initial blood pressure tended to exhibit a large increase in blood pressure during pregnancy^39^. A study in Nigeria pointed to a decline in blood pressure levels during the mid-trimester of pregnancy with a progressive increase towards term^40^. This mixed findings from the literature together with our findings suggest that the association between pregnancy and women BP is not consistent.

Our analysis also showed that women who were breastfeeding and lived in rural settings tended to have lower BP relative to those who were not breastfeeding. The negative effect of breastfeeding on BP occurred at three quantiles each for DBP (5^th^, 10^th^ and 75^th^) and SBP (50^th^, 75^th^ and 90^th^). The largest reduction in DBP and SBP occurred at the 75^th^ quantile. Breastfeeding was also inversely related to DBP in urban settings at the 25^th^ and 50^th^ quantiles. Thus, women who were breastfeeding and at these quantiles were likely to have lower SBP relative to those who were not breastfeeding. The findings confirm the widely recognized benefits of breastfeeding for improved health and developmental outcomes in mothers and their infants^41–43^. The implication of this may be that interventions to promote breastfeeding may also impact BP risk in women at the upper end of the conditional BP distribution. However, the OLS results show that breastfeeding status had a strong inverse effect on BP for all women in the sample who were breastfeeding. This is somewhat misleading, as differential effects have been observed. Relating the results of the present study to what is in the literature, one can observe some similarities. A study showed that breastfeeding resulted in lower SBP in mothers, especially at one month postpartum compared with those using other feeding modes^44^. Another study showed that both systolic and diastolic BP fell during a breastfeeding session, and pre-breastfeeding BP decreased during at least the first 6 months of a breastfeeding period in a homelike environment^41^. These studies together with the present study lend further support to the health-promoting effects of breastfeeding.

### Strengths and limitations

An important strength of the analyses is the use of large nationally representative data, thereby providing more robust estimates of observed associations. Additionally, the DBP and SBP were objectively measured, reducing possible recall bias and misclassification. The use of quantile regression in the analysis is also an important methodological strength of the paper, as previous research mostly relied on OLS, which may have misleading results. Quantile regression estimates allow for a more comprehensive picture of the effects of the factors that drive DBP and SBP. The OLS techniques summarize the average relationship between a set of regressors and the outcome variable based on the conditional mean function^31,45^, which provides only a partial view of the relationship, since we might be interested in describing the relationship at different points in the conditional distribution of the outcome variable. QR provided more detailed insights beyond the measures of central tendency (mean) and detects sources of considerable heterogeneity in the effects of explanatory variable along the entire distribution of the outcome variable^31,46^.

The cross-sectional nature of the data makes it difficult to attribute causation, and is a limitation worth mentioning. The conclusions in the paper are therefore interpreted as mere associations between the predictor variables and the outcome variables. Also, due to data limitation, we could not examine other important drivers such as genetic predisposition, family history of BP, low birth weight, smoking (only 5 cases, representing 0.05% of the total sample), alcohol consumption, and air pollution. Nevertheless, since it is almost impossible to control for all factors in a cross sectional study such as this, we are confident that the results obtained herein are robust enough to contribute to the literature and intervention planning in this area of work.

## Conclusion

This study employs quantile regression to estimate the effects of potential modifiable and non-modifiable drivers of systolic and diastolic BP among urban and rural women. The effects of the putative drivers on BP in urban and rural settings are similar. Women BMI has positive and significant effect on women BP in at least three quantiles in both urban and rural samples, with the largest effect occurring at the 75^th^ quantile, suggesting that women at this quantile are more likely to benefit more from a reduction in BMI than those at the lower end of the distribution. Systolic and diastolic BP increased with age, with the effect disproportionately accruing at the 90^th^ quantile in both urban and rural settings. Parity was inversely related to BP, with DBP and SBP decreasing with increasing parity. Women who were breastfeeding were found to have lower systolic and diastolic BP relative to those who were not breastfeeding, with largest reduction occurring at the 75^th^ quantile. This implies that interventions to promote breastfeeding may have more benefit for women BP control at the upper end of the conditional BP distribution.

### Data availability statement

This study was a re-analysis of existing data that are publicly available from The DHS Program at http://dhsprogram.com/publications/publication-fr221-dhs-final-reports.cfm. Data are accessible free of charge upon a registration with the Demographic and Health Survey program (The DHS Program). The registration is done on the DHS website indicated above.
